# Development of an On-Column Trace Enrichment Method for the Determination of Sub-*μ*g/L Bisphenol A in Bottled Water by RP-HPLC with Fluorescent Detection

**DOI:** 10.1155/2024/8258123

**Published:** 2024-01-24

**Authors:** Kevin C. Honeychurch

**Affiliations:** School of Applied Sciences, University of the West of England, Frenchay Campus, Coldharbour Lane, Bristol BS16 1QY, UK

## Abstract

A simple extraction-free, on-column trace enrichment liquid chromatographic method for the determination of trace levels of bisphenol A (BPA) in bottled water samples has been developed. It was found possible to determine ng/L (ppt) levels of BPA by the direct introduction of 6 mL of sample water to the HPLC column utilising fluorescence detection (Ex*λ* = 274 nm, Em*λ* = 314 nm). Following the loading of the sample and the chromatographic focusing of the BPA on the analytical column, a simple switch from the aqueous sample to the isocratic chromatographic elution step of 50% acetonitrile/deionised water was undertaken. Using a BPA concentration of 0.596 *μ*g/L the effect of sample volume was investigated over the range 1.0 to 12 mL. A linear relationship with the sample volume introduced to the HPLC column and the resulting peak height for BPA was found over the entire range investigated (*R*^2^ = 0.999). Using a sample volume of 6.0 mL, a well-defined chromatographic peak was recorded for BPA over the concentration range of 0.1 *μ*g/L to 6.25 *μ*g/L (*R*^2^ = 0.9998). A limit of detection of 0.058 *μ*g/L for BPA was calculated based on 3 *δ*. A mean recovery of 100% with an associated %CV of 7.6% (*n* = 5) was obtained for a bottled spring water sample fortified with 1.25 *μ*g/L BPA. Samples can be processed in under 12 minutes, much faster than that commonly reported for conventional offline extraction and chromatographic-based methods. The results show that the optimised method holds promise for the determination of BPA in such samples.

## 1. Introduction

**Figure 1:**
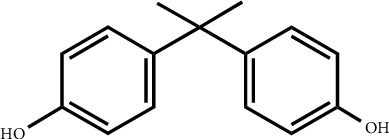
Structure of bisphenol A: 4,4′-(propane-2,2-diyl)diphenol (BPA).

Bisphenol A, or BPA, (Figure 1), is used for a number of applications such as a component in synthetic plastic materials, such as poly (vinyl chloride) and as an antioxidant in glues, plastics, and ink. However, its main application is as a monomer in the production of polycarbonate (PC), which is used for a wide range of applications. Exposure to BPA can result in harm during embryonic and foetal development and result in conditions, such as obesity, diabetes, and heart disease [1–8]. Similarly, adverse effects have been reported for both animals [9, 10] and plants [11].

An important source of human exposure is thought to be the ingestion of food and drink that has been in contact with epoxy resins or polycarbonate plastics [12, 13]. This has led to an increasing level of interest in levels of BPA that can be present in both drinking water and bottled water. In a 2009 study [14], it was shown that as little as one week of consuming cold beverages from PC bottles increased urinary BPA concentrations by two-thirds. The amount of BPA leached from PC containers is dependent on a number of parameters, such as the mineral content and pH of the water or food, the surface-to-volume ratio, the exposure time, temperature, and light [15].

The leached BPA would seem to result from the unreacted BPA monomer migrating from the PC, with the various chemical and physical processes accelerating its migration. These processes are also accelerated when reusing PC, particularly when it is damaged, or following washing and exposure to cleaning agents and hypochlorite [16, 17]. Recently, The European Food Safety Authority (EFSA) has dramatically lowered the tolerable daily intake (TDI) for BPA from 4 to 0.2 ng/kg body weight/day [18]. Accordingly, there is great interest in the development of analytical techniques which are capable of determining BPA at low trace levels in a variety of different sample matrices. High-performance liquid chromatography (HPLC) with either UV [19], electrochemical [20, 21], fluorescence detection [22], or mass spectrometry [23, 24] has been reported for the determination of BPA. Gas chromatography following derivatisation [25–27] has also been employed. Methods based on the competitive enzyme-linked immunosorbent assay (ELISA) [28] and molecularly imprinted polymers [29] have also been reported.

Due to the low concentrations commonly encountered, analytical protocols including sample preconcentration steps such as solid phase extraction (SPE) [13, 19, 30, 31] or liquid/liquid extraction [27, 32] are generally employed. Such preconcentration steps can be time-consuming, labour-intensive, suffer from varied recovery efficiencies, and require large sample volumes. This is also potentially problematic, as these sample processing steps can potentially introduce BPA contamination [33, 34].

Conventionally, in HPLC sample extracts are normally obtained by either SPE or liquid/liquid extraction [35–37]. The resulting extract is then taken down to dryness under nitrogen, following, commonly, a preconcentration step, such as rotary evaporation, and the sample residue is reconstituted in a relatively small volume of mobile phase, such as a 1.0 mL. Volumes of between 10 and 20 *μ*L of this sample extract are introduced to the HPLC column via some form of fixed-volume sample loop.

If we calculate the actual percentage of the original sample introduced to the HPLC under these conditions: we see that 20 *μ*L taken from 1.0 mL means that we are effectively only introducing 2% of the sample, and the remaining 98% is effectively wasted. For example, if a 1.0 L sample of water was taken and following extraction, concentrated to 1.0 mL, the introduction of 20 *μ*L of sample to the HPLC would be the same as introducing only 20 mL of the original sample to the instrument.

Nevertheless, one particular alternative approach that overcomes these issues is on-column trace enrichment liquid chromatography. This consists of the direct introduction of a low eluting strength solution such as the water sample itself, directly to the analytical column, where the target analytes are directly focused and concentrated. The introduction of relatively large sample volumes in the hundreds of *µ*L to mL levels [38–46] allows for the determination of low analyte concentrations without the need of time-consuming extraction steps. Following concentration of the analyte on the analytical HPLC column, the target analytes can be eluted and chromatographically separated via the introduction of the appropriate mobile phase.

This present study has focused on the determination of BPA in bottled water by HPLC single-column trace enrichment (HPLC-SCTR) with fluorescence detection. In the first part of the study, conditions such as sample volume and chromatographic conditions were optimised. The possibility of using the optimised method to determine µg/L levels of BPA in a fortified bottled water sample was then investigated by HPLC-SCTR.

## 2. Experimental

### 2.1. Chemicals and Reagents

Solids of BPA, bisphenol F, and bisphenol S were all obtained from Merck (Gillingham, UK). Acetonitrile was obtained from Fisher Scientific (Loughborough, UK). Deionised water was obtained from a Sartorius Arium® mini Ultrapure Water System (Sartorius UK Ltd., Epsom, UK). Polyethylene terephthalate bottled spring water samples (Princes Gate Water, Pembrokeshire, Wales, UK) were obtained from a local commercial outlet. A 100 mL aliquot of this was taken and fortified to be 1.25 *μ*g/L BPA.

### 2.2. Liquid Chromatography and Single-Column Trace Enrichment

**Figure 2:**
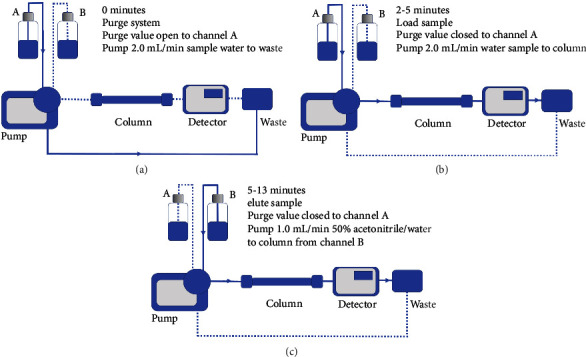
Loading, elution, and chromatographic separation of sample, in the single-column trace enrichment fluorescence detection of BPA. (a) Purging system; (b) loading of sample; (c) elution and chromatographic separation of sample.

Initial investigations were undertaken to optimise the chromatographic separation of BPA using an injection volume of 20 *μ*L. Separations were achieved using a Hypersil GOLD analytical column (150 × 4.6 mm, 5 *μ*m) with fluorescent detection using an excitation wavelength of 274 nm and an emission wavelength of 314 nm. Mobile phases with acetonitrile concentrations of between 20% and 70% in deionised water were investigated. Further, HPLC-SCTR investigations were made using a TSP Spectra SYSTEM P4000 HPLC pump (Spectra-Physics) connected directly to a Hypersil GOLD (150 × 4.6 mm, 5 *μ*m) analytical column. Fluorescence detection (linear, Fluor LC305) was undertaken using an excitation wavelength of 274 nm and an emission wavelength of 314 nm. The system was first purged with 50% acetonitrile/deionised water and then with the sample water under investigation (Figure 2(a)). Sample aliquots were directly focused on the analytical column by pumping the sample water directly to the analytical column at a flow rate of 2.0 mL/min for 3 minutes (Figure 2(b)). The focused sample components were then eluted from the analytical column by switching directly to a mobile phase of 50% acetonitrile (Fischer, far UV, HPLC grade)/deionised water at a flow rate of 1.0 mL/min (Figure 2(c)). Chromatograms were recorded using a Siemens Kompenosograph X-T C1012 chart recorder.

## 3. Results and Discussion

### 3.1. Optimisation of On-Column Trace Enrichment Liquid Chromatography

#### 3.1.1. Liquid Chromatography and Sample Loading Conditions

To optimise the chromatographic separation of BPA, initial studies were undertaken using mobile-phase organic modifier concentrations of 20%, 50%, and 70% acetonitrile/deionised water. A mobile phase of 50% acetonitrile/deionised water was found to give a good balance between resolution and runtime and was used in further investigations. The possibility of accumulating trace concentrations of BPA at the HPLC column was investigated by pumping volumes of between 1.0 and 12 mL of deionised water containing 0.596 *μ*g/L of BPA at a flow rate of 1.0 mL/min to the HPLC column (Table 1). Following loading, the flow to the column was switched directly to 50% acetonitrile/deionised water (1.0 mL/min), maintaining column pressure and overcoming possible issues of column phase collapse [47, 48], and the eluent monitored by fluorescence detection. A linear relationship between the sample volume introduced to the HPLC and the resulting peak height for BPA was found over the entire range investigated (*R*^2^=0.999).

### 3.2. Calibration Plot, Precision, Limit of Detection, and Possible Interferences

**Figure 3:**
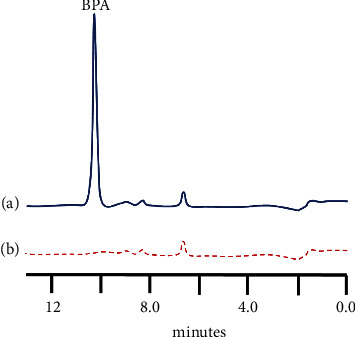
Typical chromatogram obtained for (a) 6.24 *μ*g/L BPA and (b) blank deionised water. Sampling loading for 3 minutes at 2.0 mL/min; followed by chromatographic elution at 1.0 mL/min with 50% acetonitrile/water. Fluorescence detection: Ex*λ* = 274 nm, Em*λ* = 314 nm.

**Figure 4:**
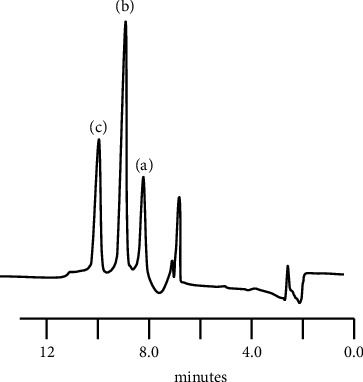
Typical chromatogram obtained for 1.20 *μ*g/L of (a) bisphenol S, (b) bisphenol F, and (c) BPA. The sample was loaded for 3 minutes at 2.0 mL/min, followed by chromatographic elution at 1.0 mL/min with 50% acetonitrile/water. Fluorescence detection: Ex*λ* = 274 nm and Em*λ* = 314 nm.

Standard solutions of 0.10, 0.625, 1.25, 2.30, 6.25, 9.40, and 12.5 *μ*g/L BPA were prepared in deionised water and examined using a loading volume of 6.0 mL using the optimised HPLC conditions.  Figure 3(a) shows the typical chromatogram obtained for a 6.24 *μ*g/L BPA standard in deionised water obtained under these conditions. No peaks were determinable for the deionised water run under same conditions (Figure 3(b)). A linear relationship was obtained over the range of 0.10 *μ*g/L to 6.25 *μ*g/L (*R*^2^=0.9998). A limit of detection, based on 3*σ*, of 0.058 *μ*g/L for BPA was found based on these values. It should be noted that both the sensitivity and limit of detection for the method could be readily improved by increasing the loading volumes employed [49].  Table 2 shows a comparison of other chromatographic methods for the determination of BPA. This illustrates the relatively large sample volumes required, which all require offline processing steps such as SPE or liquid extraction. Concentrations of 1.2 *μ*g/L of bisphenol F and bisphenol S were investigated as possible interferences and were found to be chromatographically separated from BPA and hence did not interfere (Figure 4).

### 3.3. Analytical Application

**Figure 5:**
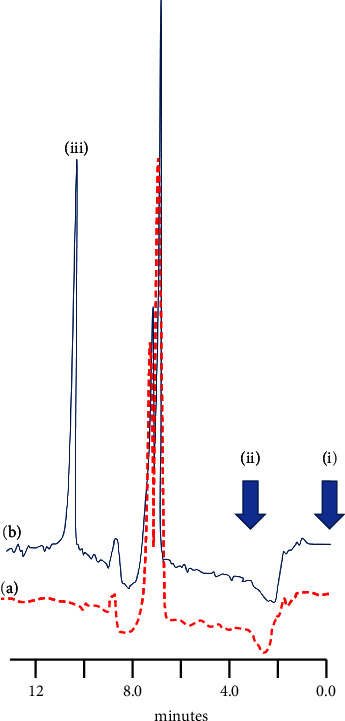
Polyethylene terephthalate bottled spring water (Pembrokeshire, Wales, UK) (a) fortified with 1.25 *μ*g/L BPA (b). Introduction of sample (i), switch to mobile phase (50% acetonitrile/deionised water) (ii); chromatographic peak for BPA (iii).

Concentrations of BPA are reported in waste and environmental water to be, in some cases, as high as the hundreds of *μ*g/L [28, 55]. However, they are generally in the sub to low *μ*g/L concentrations in drinking and bottled water [32, 56]. Consequently, to assess the performance of the optimised HPLC-SCTR approach, five replicate determinations of a bottled spring water sample fortified with BPA at a concentration of 1.25 *μ*g/L were undertaken. Quantification was undertaken through external calibration.  Figure 5 shows a representative chromatogram for this water sample fortified with 1.25 *μ*g/L BPA. A mean percentage recovery of 100% was calculated with a corresponding %CV of 7.6%. The results show that the optimised method holds promise for the determination of BPA in such samples.

## 4. Conclusions

An assay involving HPLC-SCTR with fluorescence detection has been successfully developed for the determination of trace levels of BPA in spring bottled water, at concentrations commonly previously reported. The detection method is based on the trace enrichment and direct focusing of BPA from the water sample on the analytical HPLC column. This removes the need for solvent or SPE, derivatisation steps or the need for deuterated internal standards, greatly simplifying and reducing the analysis time and expense. Samples can be processed in less than 12 minutes, obtaining performance characteristics comparable to that reported for LC/MS. In this study, a sample volume of 6.0 mL was used, allowing for a limit of detection of 0.058 *μ*g/L BPA. It should be readily simple to improve on this by increasing the volume of sample introduced.

As far as we are aware, this report is the first to describe the use of a HPLC-SCTR assay for the detection of BPA in bottled water. However, we believe that the approach developed here could form the basis of a generic approach for the analysis of other trace contaminates in drinking and bottled water, and in future studies, we plan to investigate this further. Legislation on the usage of BPA is expected to lead to a decline in its occurrence, and the presence of its analogues, bisphenol S, bisphenol F, and bisphenol AF, is likely to increase in the future. It would be readily possible to determine these compounds in water using this same approach.

## Figures and Tables

**Table 1:** Effect of loading volume on the resulting peak height for a 0.596 *μ*g/L BPA standard.

Loading volume (mL)	Fluorescence units
0	0.00
1	5.00
5	24.5
6	29.0
8	39.6
10	49.5
12	59.4

**Table 2:** Sample extraction and analytical measurement methods for the determination of BPA in water.

Sample	Sample volume (mL)	Sample extraction	Technique	Limit of detection (*μ*g/L)	Ref.
Drinking water	50	Solid-phase extraction	Ultra-performance liquid chromatography-tandem mass spectrometry	0.01	[13]
River water	50	Automated column switching	High performance liquid chromatography fluorescence detection	0.00009	[49]
Plastic bottled drinking water	3	Solid-membrane extraction based on electro spun nylon 6 nanofibrous membrane	High performance liquid chromatography	0.15	[19]
Tap, lake, and river water	20	Biosorption-based dispersive liquid-liquid microextraction	Gas chromatography/mass spectrometry (GC/MS)	0.15	[27]
Environmental and tap water	5	Ionic liquid dispersive liquid phase microextraction	High performance liquid chromatography mass spectrometry	0.76	[50]
Effluents, soil leachates, food, drinking water and consumer products	—	—	Competitive enzyme-linked immunosorbent assay (ELISA)	5	[28]
Water exposed to polycarbonate animal cages	220	Extracted with dichloromethane in separating funnel	Gas chromatography/mass spectrometry (GC/MS)	0.05–0.1	[51]
Environmental and tap water	1000	Solid-phase extraction	Ultra performance convergence chromatography	10	[30]
Urine, drinking, river and wastewater	20	Magnetic dispersive solid-phase extraction	High performance liquid chromatography with diode array detection	0.6	[52]
Polycarbonate bottled water	25	Solid-phase extraction	High performance liquid chromatography with fluorescence detection	0.30	[31]
Plastic bottled drinking water	0.02	None, direct injection of water sample	High performance liquid chromatography with UV detection	380	[53]
Narrowly dispersible imprinted polymeric microspheres columns	40	None	High performance liquid chromatography single-column trace enrichment UV detection	22.8	[54]
Plastic bottled drinking water	6	None	High performance liquid chromatography single-column trace enrichment fluorescence detection	0.058	This study

## Data Availability

The data used to support the findings of this study are included within the article.

## Supplementary Materials

Supplementary Materials Supplementary material Graphical Abstract.
