# Supplementary figures and images for: Development of an On-Column Trace Enrichment Method for the Determination of Sub-μg/L Bisphenol A in Bottled Water by RP-HPLC with Fluorescent Detection
Source: Int J Anal Chem. 2024 Jan 24;2024:8258123. doi: 10.1155/2024/8258123 (PMC10831038; doi:10.1155/2024/8258123)

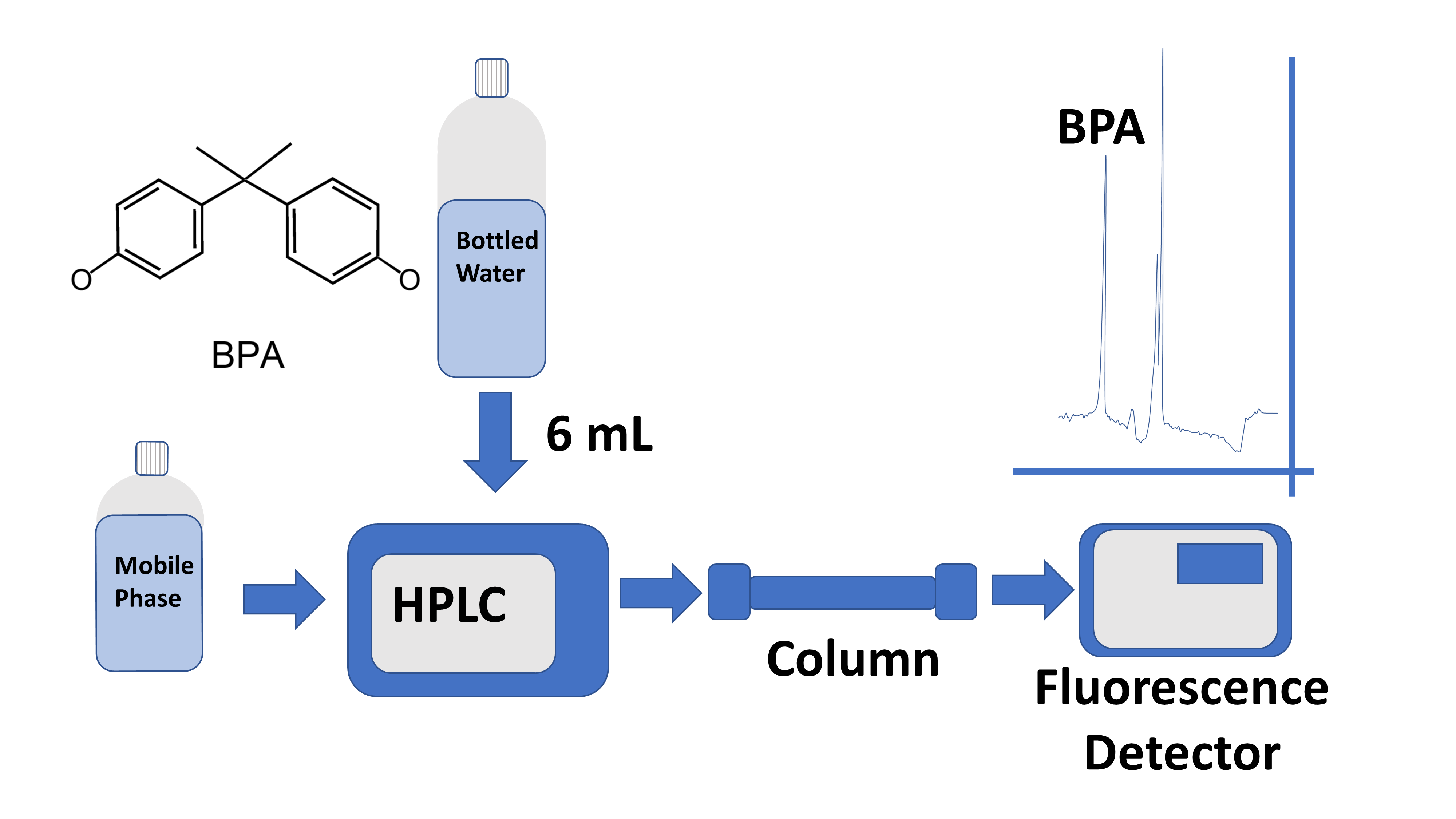

Supplement: Supplementary Materials — Supplementary material Graphical Abstract. [file 8258123.f1.png]
